# Early fruiting in *Synsepalum dulcificum* (Schumach. & Thonn.) Daniell juveniles induced by water and inorganic nutrient management

**DOI:** 10.12688/f1000research.11091.1

**Published:** 2017-03-30

**Authors:** Dèdéou Apocalypse Tchokponhoué, Sognigbé N'Danikou, Iago Hale, Allen Van Deynze, Enoch Gbènato Achigan-Dako

**Affiliations:** 1 Laboratory of Genetics, Biotechnology and Seed Sciences, University of Abomey-Calavi, Abomey-Calavi, Benin; 2 Department of Biological Sciences, University of New Hampshire, Durham, NH, 03824, USA; 3 Seed Biotechnology Center, University of California, Davis, CA, 95616, USA; 4 Department of Plant Sciences, University of California, Davis, CA, 95616, USA

**Keywords:** Mineral fertilization, juvenility phase, precocity, environmental induction, growth, flowering, miracle berry

## Abstract

**Background.** The miracle plant, *Synsepalum dulcificum* (Schumach. & Thonn.) Daniell is a native African orphan crop species that has recently received increased attention due to its promise as a sweetener and source of antioxidants in both the food and pharmaceutical industries. However, a major obstacle to the species’ widespread utilization is its relatively slow growth rate and prolonged juvenile period. **Method.** In this study, we tested twelve treatments made up of various watering regimes and exogenous nutrient application (nitrogen, phosphorus and potassium, at varying dosages) on the relative survival, growth, and reproductive development of 15-months-old *S. dulcificum* juveniles. **Results.** While the plants survived under most tested growing conditions, nitrogen application at doses higher than 1.5 g [seedling] ^-1 ^was found to be highly detrimental, reducing survival to 0%. The treatment was found to affect all growth traits, and juveniles that received a combination of nitrogen, phosphorus, and potassium (each at a rate of 1.5 g [seedling] ^-1^), in addition to daily watering, exhibited the most vegetative growth. The simple daily provision of adequate water was found to greatly accelerate the transition to reproductive maturity in the species (from >36 months to an average of 23 months), whereas nutrient application affected the length of the reproductive phase within a season, as well as the fruiting intensity. **Conclusions.** This study highlights the beneficial effect of water supply and fertilization on both vegetative and reproductive growth in *S. dulcificum*. Water supply appeared to be the most important factor unlocking flowering in the species, while the combination of nitrogen, phosphorus and potassium at the dose of 1.5 g (for all) consistently exhibited the highest performance for all growth and yield traits. These findings will help intensify *S. dulcificum*’s breeding and horticultural development.

## Introduction

The miracle plant, *Synsepalum dulcificum* (Schumach. & Thonn.) Daniell (Sapotaceae), is a perennial shrub originating from West Africa ( Inglett & May, 1968) and is the only known natural source of miraculin, a glycoprotein with remarkable edulcoration properties ( Lim, 2013). In West Africa, the sweetening activity of the fruit is valued in drink-making, whereas the leaves, roots, and bark of the species are used in traditional treatments of diabetes, enuresis, kidney, hyperthermia, coughing, and stomach afflictions ( Burkill, 2000; Oumorou *et al.*, 2010). The fruit of the species (miracle berry) is a rich source of vitamin C, leucine, flavonols, and anthocyanin ( Du *et al.*, 2014; Njoku *et al.*, 2015); and its modern utilizations include many applications in cosmetics, food, and pharmaceuticals ( Achigan-Dako *et al.*, 2015). With its many unique properties, some writers have suggested that miracle berry would currently have a much larger market in the USA, and therefore globally, if it had not been misclassified in the 1970's as a food additive instead of a sweetener ( http://www.gayot.com/Lifestyle/Health/Benefits/Miracle-Fruit; http://www.theweek.co.uk/politics/27131/sweet-and-sour-tale-miracle-berry). Recently, additional scientific evidences were highlighted on the ability of the species to substitute sugar, particularly in sour beverages ( Rodrigues *et al.*, 2016).

Despite the nutritional, economic, and medicinal promise of the species, *S. dulcificum* remains a neglected crop that is not widely cultivated. In addition, according to Adomou (2005), the species is in depletion and is also suspected to exhibit recalcitrant seed storage behavior ( Chen *et al.*, 2012). One of the major constraints to economic cultivation of miracle berry is the very slow growth rate and the prolonged juvenile phase of the plant. According to Joyner (2006), the species seedling size at four years old is a maximum of 60 cm and fructification occurs only after three to four years; however, information regarding the growing conditions of the seedlings in that study was lacking. In Benin, where the plants are also reported to exhibit a relatively slow growth rate and to be late maturing, the species is almost wholly neglected. When encountered in its natural habitat (open field), the species exhibits relatively poor fitness in the face of weed competition, as well as anthropogenic and animal disturbances ( Houeto, 2015).

An important step toward the systematic improvement of *S. dulcificum* would be to accelerate the transition to reproductive maturity, thus shortening generation times. According to Wilkie *et al.* (2008), there are three possible ways to induce flowering in horticultural trees, thereby reducing the length of the juvenile phase, or increasing precocity: environmental induction, autonomous induction, and the use of growth regulators. A plant’s ability to favorably respond to any of these flowering induction techniques greatly depends on its origin. While tropical and subtropical species tend to respond better to environment stimuli (e.g. mango, *Mangifera indica* L.; lychee, *Litchi chineensis* Sonn.), those from temperate regions exhibit autonomous floral induction (e.g. apple, *Malus domestica* Borkh.; sweet cherry, *Prunus avium* L.) ( Wilkie *et al.*, 2008). Given that *S. dulcificum* is a tropical species, we hypothesize that an accelerated transition to reproductive maturity can be triggered through proper environmental manipulation. Additionally, in woody angiosperms, cold treatment, nutrient supply, photoperiod, and water stress were found to be the main environmental stimulations that could induce flowering ( Meilan, 1997).

One important factor limiting plant growth is nitrogen and phosphorus deficiency ( King *et al.*, 2008; Poothong & Reed, 2014). Nutrient status has been reported to affect gene activity and protein synthesis in plant species (e.g. Japanese red pine, *Pinus densiflora* Sieb. & Zucc.) ( Nakaji *et al.*, 2001). For instance, a high C/N ratio was reported to favor flowering in fruit trees ( Hanke *et al.*, 2007). Fertilization management thus appears to be a promising means of promoting plant growth and early flowering in horticultural species; and yet, different plant species tend to react to nutrient supply in unpredictable ways. For example, while phosphorus fertilization was found to be beneficial for the lobolly pine ( *Pinus taeda* L.) growth, nitrogen fertilization on the same species was rather detrimental ( Faustino *et al.*, 2013). In another study, phosphorus fertigation was shown to be harmful to the fan flower ( *Scaevola aemula* R. Br.) when applied at a rate exceeding 43.5 g.ml ^-1^ ( Zhang *et al.*, 2004). In many other species, such as marula, *Sclerocarya birrea* (Hochst.) and wild loquat, *Uapaca kirkinia* (Muell.Arg.), the benefit of fertilizer application remains elusive ( Akinnifesi *et al.*, 2008). Similarly, water availability is considered to be one of the three most important factors controlling a plant's transition to flowering ( Bernier *et al.*, 1993), in addition to affecting the phenological rhythm of tropical species; and yet plant response to water stress (excess/deficiency) also tends to be species-specific. While water deficiency was found to promote flowering in *Citrus* spp. ( Davenport, 2003), it reduced vegetative growth in *Mangifera indica* L. ( Pavel & De villiers, 2004).

To the best of our knowledge, the response of *S. dulcificum* to fertilization and regular water supply has never been documented. Furthermore, detailed phenological data, especially in juveniles, are not available despite their importance to pioneering breeding programs. Understanding how nutrient and water supply affect fruiting in *S. dulcificum* juveniles is critical to the development of this promising species.

In this study, we analyzed the growth, flowering, and fruiting response of *S. dulcificum* to water and mineral fertilizer treatments with the objective of reducing the species natural (in reference to stands evolving in natural habitat) production cycle, while significantly enhancing overall growth and fruit yield.

## Methods

### Experimental site

The experiment was carried out from December 2013 to April 2016 in the municipality of Abomey-Calavi (southern Benin), at the experimental site of the Faculty of Agronomic Sciences, University of Abomey-Calavi (06°25’00.8”N, 002°20’24.5”E), and in a neighboring open field (06°27’00.”N, 002°21’00”E) to simulate natural rain fed conditions (no irrigation or exogenous nutrient application). Abomey-Calavi is located in the Guinean phytogeographical region of Benin largely characterized by a ferralitic soil type ( Röhrig, 2008). During the experimental timeframe, the mean annual rainfall was 1,329 mm and the mean monthly temperature was 24°C.

### Experimental system

In December 2013, mature, ripe and fresh fruits of *S. dulcificum* were collected from a single tree located in the district of Toffo (6°92’N; 2°27’E), where the soil is ferralitic, the mean annual rainfall is around 1,000 mm, and the mean annual temperature varies from 27°C to 30°C. Fruits collected were processed and sown at ambient temperature (25–27°C) in black polystyrene nursery bags (0.75 l) filled with sand to produce seedlings that were monitored in the nursery until they reached 13 months old. At that time, seedlings of a similar size were transplanted either in pots on the site of University of Abomey-Calavi or directly at soil in the open field and monitored for two months before being used in the watering and fertilization experiment. There was only one seedling per pot and each pot had 15 l volume.

The experiment was made up of twelve treatments ( Table 1), out of which the absolute control (Cont: rain fed seedlings with no nutrient supply) was established at soil in the open site and the other 11 treatments were established in pots (to control the amount of water supply and its efficiency) filled with soil collected at 0–10 cm depth on the site of University of Abomey-Calavi. Each seedling in pots received two liters of water daily. Nutrients were brought to each pot (seedlings) separately; the nitrogen was applied as urea (46% N), the phosphorus as simple superphosphate (46% P _2_O _5_) and the potassium as potassium sulfate (48% K _2_O). Fertilizers were applied using the sub-surface method at 8 cm beneath the soil and at a frequency of one application every two months. The first application occurred in March 2015. Physico-chemical characteristics of the experimental medium in pots were as follows: pH (KCl) = 5.48, pH (H _2_O) = 5.88, silt = 25.75%, clay = 12.27%, sand = 61.98%, organic carbon = 1.03%, N = 0.06%, Mg = 2.37 (meq/100g), Ca = 0.63 (meq/100g), P = (2.08 meq/100g), and assimilable P = 23.06 ppm. The experiment design was of completely randomized design and each treatment was made up of a cohort of 10 seedlings of the same age (15 months). We used this sample size because *S. dulcificum* is a recalcitrant perennial, and obtaining progeny individuals of similar age and size was challenging.

**Table 1.** Treatments, amount of water supplied and nutrient doses applied at each fertilization event.

Treatments	Daily watering (l.seedling ^-1^)	N (g. seedling ^-1^)	P (g. seedling ^-1^)	K (g. seedling ^-1^)
Control	-	-	-	-
W	2	-	-	-
N1.5	2	1.5	-	-
N3	2	3.0	-	-
N4.5	2	4.5	-	-
P1.5	2	-	1.5	-
P3	2	-	3.0	-
P4.5	2	-	4.5	-
K1.5	2	-	-	1.5
K3	2	-	-	3.0
K4.5	2	-	-	4.5
NPK	2	1.5	1.5	1.5

### Data collection

***Measuring growth parameters*.** Before treatment application, initial stem collar diameter, plant height, number of branches, and number of leaves were measured for all seedlings ( Table 2) to ensure that seedlings had similar size. At the end of the experiment (April 2016), the same traits were also measured to evaluate the increments.

**Table 2.** Initial growth parameters in juveniles of *Synsepalum dulcificum* at experiment onset. Values are means ± SE (n = 10 seedlings).

Treatments	Stem collar diameter (mm)	Height (cm)	Number of leaves	Branching
Cont	4.28 ± 0.29 ^a^	16.33 ± 1.35 ^a^	40.2 ± 4.36 ^a^	5.30 ± 0.21 ^a^
W	3.97 ± 0.41 ^a^	14.08 ± 1.16 ^a^	36.30 ± 8.31 ^a^	4.50 ± 0.5 ^a^
N1.5	3.91 ± 0.32 ^a^	16.54 ± 0.92 ^a^	39.20 ± 6.32 ^a^	4.90 ± 0.43 ^a^
N3	3.96 ± 0.37 ^a^	15.84 ± 1.33 ^a^	41.80 ± 7.24 ^a^	5.00 ± 0.59 ^a^
N4.5	4.30 ± 0.33 ^a^	16.58 ± 1.29 ^a^	46.10 ± 8.11 ^a^	5.00 ± 0.74 ^a^
P1.5	4.12 ± 0.27 ^a^	16.84 ± 1.62 ^a^	42.80 ± 6.95 ^a^	5.30 ± 0.53 ^a^
P3	4.41 ± 0.33 ^a^	15.72 ± 1.45 ^a^	44.20 ± 7.42 ^a^	5.20 ± 0.48 ^a^
P4.5	3.98 ± 0.33 ^a^	17.03 ± 1.57 ^a^	39.10 ± 5.64 ^a^	5.50 ± 0.71 ^a^
K1.5	4.23 ± 0.23 ^a^	16.36 ± 1.13 ^a^	37.60 ± 5.68 ^a^	4.60 ± 0.5 ^a^
K3	4.00 ± 0.27 ^a^	14.40 ± 1.51 ^a^	35.30 ± 7.22 ^a^	4.40 ± 0.8 ^a^
K4.5	4.38 ± 0.28 ^a^	17.90 ± 1.42 ^a^	45.10 ± 6.51 ^a^	5.30 ± 0.21 ^a^
NPK	4.31 ± 0.25 ^a^	18.38 ± 0.92 ^a^	47.4 ± 7.22 ^a^	4.80 ± 0.35 ^a^
P-value	**0.97 ^ns^**	**0.57 ^ns^**	**0.97 ^ns^**	**0.94 ^ns^**

ns= Not significant at 5%.

Leaf area was measured following the method by Cornelissen *et al.* (2003). The most mature and fully sun exposed leaf was harvested from each seedling. Harvested leaves were photocopied on paper, which were cut-out and weighed according to the shape of the leaves. The weight of the cut-out paper was multiplied by the known area/weight ratio of the paper to get the leaf area. Growth was assessed based on the increment recorded for each vegetative growth parameter between the onset and the end of the experiment.

Initial growth parameters at the fertilization experiment onset Click here for additional data file. Copyright: © 2017 Tchokponhoué DA et al. 2017 Data associated with the article are available under the terms of the Creative Commons Zero "No rights reserved" data waiver (CC0 1.0 Public domain dedication).

**Figure 1.**
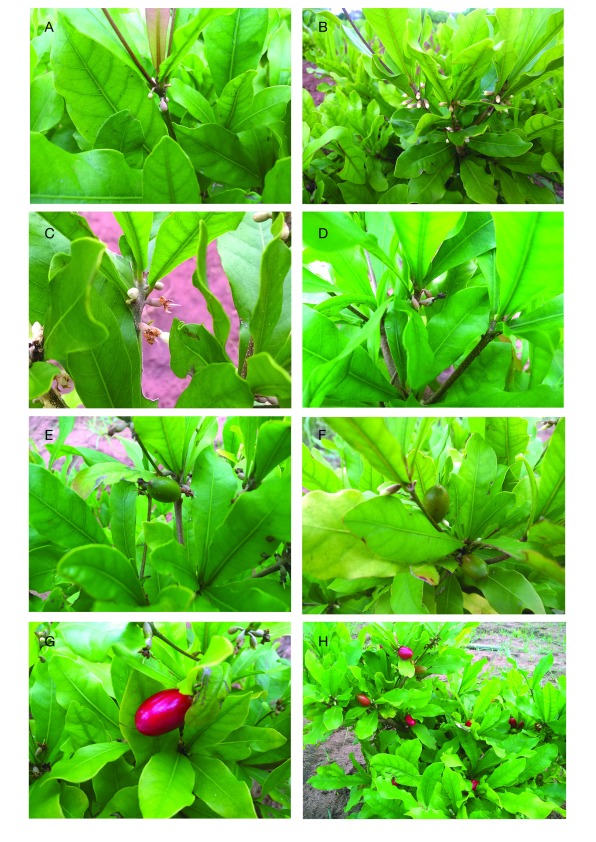
Main generative phases observed in *Synsepalum dulcificum* juveniles. ( **A**) Budding; ( **B**) Flowering; ( **C**) Flower bloom; ( **D**) Fructification onset; ( **E**) Physiological maturing; ( **F**) Fruit ripening onset; ( **G**) and ( **H**) Fruit full ripening. **S _1_** A→B; **S _2_** B→C; **S _3_** C→D; **S _4_** D→E; **S _5_** E→F; **S _6_**F→G, H.

***Tracking flowering phases*.** From the first day of treatment application to the end of experiment, we monitored each seedling development daily. Within the so-called generative phase, starting with budding and ending with fruit ripening, we distinguished seven main events (budding, flowering, flower bloom, fructification onset, fruit physiological maturity, ripening onset, and full ripening) demarcating six distinct phases (S _1_: budding to flowering, S _2_: flowering to flower bloom, S _3_: flower bloom to fructification onset, S _4_: fructification onset to physiological maturity, S _5_: physiological maturity to fruit ripening onset, and S _6_: fruit ripening onset to full ripening; see Figure 1). The occurrence date of each event was recorded and the total number of buds, flowers, and fruits per seedling were counted. The number of buds and the number of flowers were monitored until the tenth month (to avoid flower drop) of the experiment (December 2015) and only the fruiting was monitored to the end of the experiment (April 2016).

### Statistical analysis

Prior to analysis we explored the datasets, and outliers were identified using the boxplot approach ( Crawley, 2007). These outliers contained in Datasets 3 and 4 (( Tchokponhoué *et al.*, 2017c; Tchokponhoué *et al.*, 2017d) were removed from further vegetative growth analysis. Following this approach, outliers are considered as more than 1.5 times the interquartile range above the third quartile and below the first quartile. To test the effects of treatments on seedling survival, we performed a survival analysis. To analyze stem collar diameter, height, and leaf area variation in response to treatments, we performed analyses of variance followed by Tukey *post hoc* test for means separation. We employed orthogonal contrasts to dissect the effect of daily watering, as well as to analyze trends in growth response to progressive doses of nutrients when significant effects were observed. To analyze how the treatments affected the proportion of plants bearing buds, flowers, and fruits, we used prop.test. The number of branches, the number of leaves, the length of each generative phase, the number of buds, the number of flowers and the number of fruits were analyzed using a generalized linear model (glm) with poisson error structure (or quasi error structure to account for over-dispersion) where necessary. Apart from survival analysis, other statistical analyses were only performed for treatments that had at least two surviving seedlings at the end of the experiment. Also, since all seedlings considered in vegetative growth have not reached reproductive stages (e.g. budding, flowering), there is a discrepancy in the number of seedling between vegetative and reproductive growth datasets. Analyses were performed using “agricolae”, “car”, “gvlma”, ‘‘multcomp’’ and ‘‘survival’’ packages in R version 2.15.3 ( R Developement Core Team, 2013) and results are presented as means ± standard errors (SE).

## Results

### Effect of treatments on the survival of seedlings

**Figure 2.**
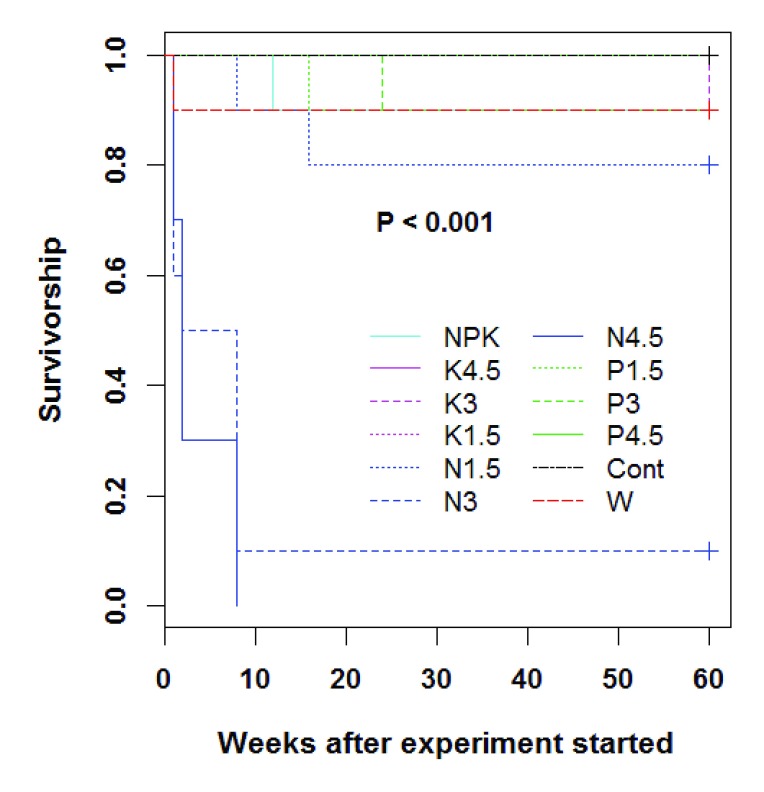
Survival trends for *Synsepalum dulcificum* juveniles under various treatments (n =10 seedlings). Cont = rain fed, no exogenous nutrients; W = Daily watering, no exogenous nutrients; N1.5 = Daily watering + 1.5 g N [seedling] ^-1^; N3 = Daily watering +3 g N [seedling] ^-1^; N4.5 = Daily watering + 4.5 g N [seedling] ^-1^; P1.5 = Daily watering + 1.5 g P [seedling] ^-1^; P3 = Daily watering + 3 g P [seedling] ^-1^; P4.5 = Daily watering + 4.5 g P [seedling] ^-1^; K1.5 = Daily watering +1.5 g K [seedling] ^-1^; K3 = Daily watering + 3 g K [seedling] ^-1^; K4.5 = Daily watering + 4.5 g K [seedling] ^-1^; NPK = Daily watering + 1.5 g N + 1.5 g P + 1.5 g K [seedling] ^-1^.

At the end of the experiment, the survival rate in the juveniles was highly affected by the treatment ( *P <* 0.001), with the lowest survival rates observed in nitrogen-based treatments ( Table 3). For this specific nutrient type (N), the higher the dose, the lower the survival and the more abrupt the survival decline. For instance, while the average time to death in juveniles that received 1.5 g nitrogen each was 12.00 ± 0.5 weeks, times to death in juveniles that received 3.0 g and 4.5 g nitrogen were 4.22 ± 0.3 weeks and 3.50 ± 0.3 weeks, respectively ( Figure 2).

**Table 3.** Proportion and number of surviving seedling at the end of the experiment (n = 10 seedlings).

Treatments	Surviving seedlings (%)	Number of surviving seedlings
Cont	100 ^a^	10
W	90 ^a^	9
N1.5	80 ^a^	8
N3	10 ^b^	1
N4.5	00 ^c^	0
P1.5	90 ^a^	9
P3	90 ^a^	9
P4.5	100 ^a^	10
K1.5	100 ^a^	10
K3	90 ^a^	9
K4.5	100 ^b^	10
NPK	90 ^a^	9
P-value	<0.001***	**-**

Means with different letters within a column denote significant differences. ***= Significant at 1‰

Survival data Click here for additional data file. Copyright: © 2017 Tchokponhoué DA et al. 2017 Data associated with the article are available under the terms of the Creative Commons Zero "No rights reserved" data waiver (CC0 1.0 Public domain dedication).

### Vegetative growth response to treatments

The survival data indicated a survival rate less than 20% in treatments N3 and N4.5; consequently they were discarded from subsequent analyses.

**Figure 3.**
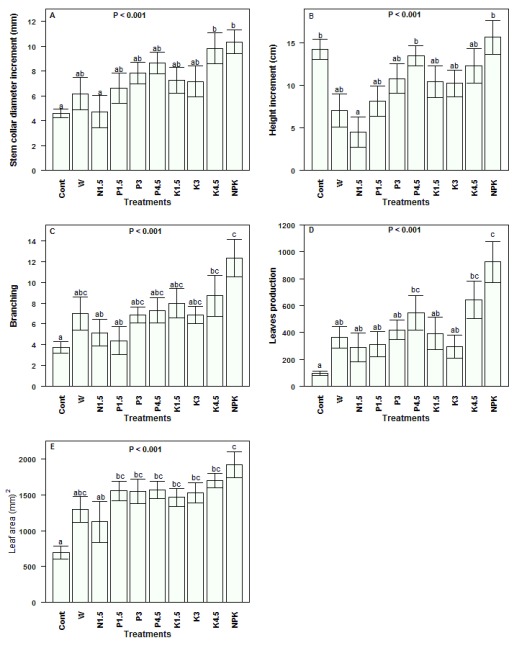
Vegetative growth response of *Synsepalum dulcificum* juveniles under various treatments. ( **A**) Stem collar diameter; ( **B**) Height; ( **C**) Branching; ( **D**) Leaf production and ( **E**) Leaf area. Values are means ± SE (n = 8 – 10 seedlings). Means with different letters denote significant differences at P < 0.05, ANOVA, Tukey Test. Cont = rain fed, no exogenous nutrients; W = Daily watering, no exogenous nutrients; N1.5 = Daily watering + 1.5 g N [seedling] ^-1^; N3 = Daily watering +3 g N [seedling] ^-1^; N4.5 = Daily watering + 4.5 g N [seedling] ^-1^; P1.5 = Daily watering + 1.5 g P [seedling] ^-1^; P3 = Daily watering + 3 g P [seedling] ^-1^; P4.5 = Daily watering + 4.5 g P [seedling] ^-1^; K1.5 = Daily watering +1.5 g K [seedling] ^-1^; K3 = Daily watering + 3 g K [seedling] ^-1^; K4.5 = Daily watering + 4.5 g K [seedling] ^-1^; NPK = Daily watering + 1.5 g N + 1.5 g P + 1.5 g K [seedling] ^-1^.

***Stem collar diameter, plant height, and branching*.** The increment in the seedlings stem collar diameter was highly affected by treatment ( Figure 3A). The daily watered juveniles performed better than the rain fed ones ( *P <* 0.001). The extent of the stem collar diameter growth also greatly differed among nutrient types. For instance, the average increment in juveniles fertilized with NPK (10.36 ± 0.96 mm) was nearly twofold higher than that in juveniles fertilized with nitrogen only (4.73 ± 1.31 mm). The stem collar diameter growth with phosphorus was as good as potassium ( *P =* 0.52), but higher than N ( *P =* 0.007), and lower than with NPK ( *P =* 0.04). We observed a highly significant effect of treatment on plant height ( Figure 3B). Contrast analysis indicated that combined N, P and K application increased plant height better than single nutrient application ( *P =* 0.01). Plants also better responded to phosphorus or potassium supply than to nitrogen ( *P =* 0.002). Meanwhile, rain fed seedlings grew taller than daily watered plants receiving a single nutrient ( *P < 0.01*).

The branching intensity also greatly varied following treatments ( Figure 3C). The average branches gain in rain fed seedlings was 3.75 *±* 0.53, whereas the set of daily watered juveniles gained on average nearly double (7.33 *±* 1.35; *P <* 0.001). The effect of nutrient supply was also significant ( *P <* 0.001) on the seedling branching, with plants fertilized with NPK gaining on average 12.33 ± 1.8 branches against 6.74 ± 1.25 for plants fertilized with a single nutrient.

***Increase in leaf number and size*.** The variation in leaf production based on treatment is presented in Figure 3D. The differences in the increment of the number of leaves due to water supply and to exogenous nutrient application were all highly significant ( *P <* 0.001). Grouped together, daily watered juveniles produced on average fourfold more leaves than rain fed juveniles. Regarding the fertilizer type, daily watered juveniles fertilized with NPK gained on average 925 *±* 154 leaves, representing for instance 2.51 times the average leaf gain in simply watered juveniles without exogenous nutrient (W). Furthermore, NPK particularly improved leaf production comparatively to single nutrient application ( *P <* 0.001). Likewise, the treatment significantly affected the leaf size with daily watered juveniles presenting a larger leaf area (1539.06 *±* 55.46 *mm ^2^*) than rain fed juveniles (695.37 *± 86.87 mm ^2^*), and leaf area in juveniles fertilized with NPK was greater than those of juveniles fertilized with a single nutrient ( Figure 3E). However, the juveniles responded better when P or K was supplied than when N was supplied.

Growth parameters (increment) at the end of the experiment for vegetative growth Click here for additional data file. Copyright: © 2017 Tchokponhoué DA et al. 2017 Data associated with the article are available under the terms of the Creative Commons Zero "No rights reserved" data waiver (CC0 1.0 Public domain dedication).

Growth parameters at the end of the experiment for leaf area Click here for additional data file. Copyright: © 2017 Tchokponhoué DA et al. 2017 Data associated with the article are available under the terms of the Creative Commons Zero "No rights reserved" data waiver (CC0 1.0 Public domain dedication).

### Flowering and fruiting responses

**Figure 4.**
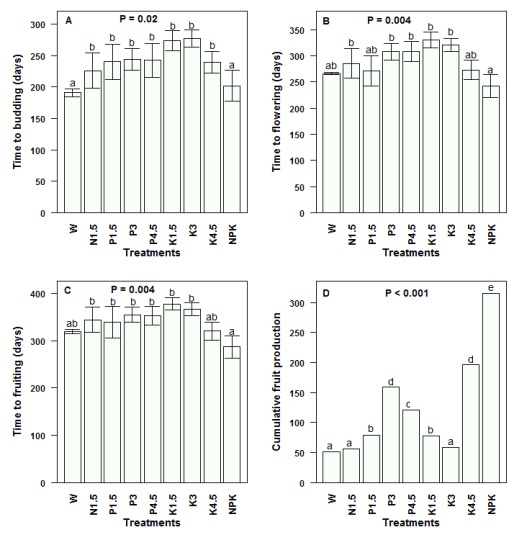
Reproductive performance in juveniles of *Synsepalum dulcificum* under various treatments. ( **A**) Time to budding; ( **B**) Time to flowering; ( **C**) Time to fruiting and ( **D**) Total fruit production. Values are means ± SE (n = 5 – 10 seedlings). Means/Values with different letters denote significant differences. Generalized linear model, Tukey Test. W = Daily watering, no exogenous nutrients; N1.5 = Daily watering + 1.5 g N [seedling] ^-1^; N3 = Daily watering +3 g N [seedling] ^-1^; N4.5 = Daily watering + 4.5 g N [seedling] ^-1^; P1.5 = Daily watering + 1.5 g P [seedling] ^-1^; P3 = Daily watering + 3 g P [seedling] ^-1^; P4.5 = Daily watering + 4.5 g P [seedling] ^-1^; K1.5 = Daily watering +1.5 g K [seedling] ^-1^; K3 = Daily watering + 3 g K [seedling] ^-1^; K4.5 = Daily watering + 4.5 g K [seedling] ^-1^; NPK = Daily watering + 1.5 g N + 1.5 g P + 1.5 g K [seedling] ^-1^.

***Budding and flowering*.** The proportion of budding juveniles was significantly affected by the treatment and ranged from 0–100% ( Table 4). The contrast analysis on the average time to budding revealed a significant effect of treatment ( *P =* 0.02; Figure 4A). Though the shortest times to budding, 190 ± 5.92 days and 201 ± 24.51 days were recorded in daily watered unfertilized juveniles and in daily watered and NPK fertilized juveniles, respectively, the highest number of buds was observed in juveniles fertilized with NPK ( Table 5). After 10 months, NPK-fertilized seedlings produced a significantly greater number of buds than unfertilized plants (six times, *P =* 0.05).

**Table 4.** Proportion and number of budding, flowering and fruiting juveniles of *Synsepalum dulcificum* based on treatments (n = 8 – 10 seedlings).

Treatments	Budding seedlings (%)	Flowering seedlings (%)	Fruiting seedlings (%)	Budding seedlings (n)	Flowering seedlings (n)	Fruiting seedlings (n)
Cont	0.00 ^d^	0.00 ^c^	0.00 ^e^	0	0	0
W	33.33 ^c^	33.33 ^b^	22.22 ^d^	3	3	2
N1.5	62.50 ^b^	50.00 ^b^	50.00 ^c^	5	4	4
P1.5	66.66 ^b^	55.55 ^b^	55.55 ^c^	6	5	5
P3	88.88 ^a^	88.88 ^a^	88.88 ^b^	8	8	8
P4.5	70.00 ^b^	70.00 ^b^	60.00 ^c^	7	7	6
K1.5	60.00 ^b^	60.00 ^b^	60.00 ^c^	6	6	6
K3	88.88 ^a^	88.88 ^a^	88.88 ^b^	8	8	8
K4.5	100.00 ^a^	100.00 ^a^	100.00 ^a^	10	10	10
NPK	100.00 ^a^	100.00 ^a^	100.00 ^a^	9	9	9
P-Value	^<^ 0.001***	^<^ 0.001***	^<^ 0.001***	**-**	**-**	**-**

Means with different letters within a column denote significant differences. ***= Significant at 1‰.

**Table 5.** Average numbers of buds and fruits produced by juveniles of *Synsepalum dulcificum* under various treatments. Values are means ± SE (n = 3 – 10 seedlings).

Treatments	Number of buds ^$^	Number of fruit ^€^
W	30.66 ± 15.05 ^b^	17.00 ± 6.24 ^b^
N1.5	43.60 ± 10.47 ^b^	25.50 ± 8.43 ^b^
P1.5	67.83 ± 62.47 ^b^	17.80 ± 12.61 ^b^
P3	38.62 ± 19.79 ^b^	19.87 ± 5.93 ^b^
P4.5	24.71 ± 13.79 ^b^	24.20 ± 5.69 ^b^
K1.5	11.5 ± 6.73 ^b^	13.00 ± 3.34 ^b^
K3	22.62 ± 20.18 ^b^	29.50 ± 18.5 ^b^
K4.5	74.10 ± 31.42 ^b^	21.88 ± 6.95 ^b^
NPK	187.55 ± 84.95 ^a^	52.50 ± 15.64 ^a^
P-value	0.05*	0.01*

^$^: assessed at the tenth month of the experiment, ^€^: assessed at the end of the experiment (thirteenth month of the experiment). Means with different letters within a column denote significant differences. *= Significant at 5%.

The proportion of flowering juveniles was also highly affected by the treatment ( Table 4). The highest flowering percentages were observed in NPK-fertilized juveniles and those fertilized with potassium at 4.5 g [juvenile] ^-1^ (100%). The time to flowering ( Figure 4B) was shorter for NPK-fertilized juveniles ( *P =* 0.004), which flowered after 242.0 ± 21.97 days compared to 299.65 ± 7.41 days for the set of single-nutrient fertilized juveniles. Within the potassium-based treatments, the effect of application dose was significant ( *P =* 0.01) and the time to flowering decreased as the potassium dose increased with a quadratic relationship between the two variables ( *P =* 0.02). The regression equation reads: ***Time to flowering = 300.52 + 49.32 Potassium dose -19.51 (Potassium dose) ^2^***.

***Fructification*.** The proportion of fruiting juveniles ranged from 0% in rain fed juveniles to 100% in NPK-fertilized plants and was highly affected by the treatment ( Table 4). Likewise, the time to fruiting in *S. dulcificum* juveniles significantly differed among treatments ( *P =* 0.004) and varied from 286 ± 9.33 days to 377 ± 5.43 days ( Figure 4C). The earliest fruiting individuals included NPK-fertilized plants. Here also, the time to fruiting was affected by the potassium dose ( *P =* 0.02). We also observed a significant quadratic relationship between the time to fruiting and the potassium application dose ( *P =* 0.03). ***The equation reads: Time to fruiting = 355.48 + 39.18 Potassium dose -16.99 (Potassium dose) ^2^***.

Furthermore, the highest cumulative fruit number per treatment ( Figure 4D) and average fruit number yielded by each plant ( Table 5) were observed in NPK-fertilized juveniles. For instance, NPK-fertilized juveniles produced twofold more fruits than those that received a single nutrient (N or P or K) and threefold more fruits than juveniles that received no nutrients ( Table 5). The fruit mass significantly differed among treatments ( *P =* 0.01) and ranged from 1.08 ± 0.17 g (in juveniles fertilized with 1.5 g phosphorus) to 1.47 ± 0.04 g (in juveniles fertilized with 3 g phosphorus).

Dataset 5. Reproductive performance (time to budding) http://dx.doi.org/10.5256/f1000research.11091.d155627
This dataset was used to prepare Figure 4A and to perform related analysis. Click here for additional data file. Copyright: © 2017 Tchokponhoué DA et al. 2017 Data associated with the article are available under the terms of the Creative Commons Zero "No rights reserved" data waiver (CC0 1.0 Public domain dedication).

Reproductive performance (time to flowering) Click here for additional data file. Copyright: © 2017 Tchokponhoué DA et al. 2017 Data associated with the article are available under the terms of the Creative Commons Zero "No rights reserved" data waiver (CC0 1.0 Public domain dedication).

Reproductive performance (time to fruiting) Click here for additional data file. Copyright: © 2017 Tchokponhoué DA et al. 2017 Data associated with the article are available under the terms of the Creative Commons Zero "No rights reserved" data waiver (CC0 1.0 Public domain dedication).

Cumulative fruiting Click here for additional data file. Copyright: © 2017 Tchokponhoué DA et al. 2017 Data associated with the article are available under the terms of the Creative Commons Zero "No rights reserved" data waiver (CC0 1.0 Public domain dedication).

Budding intensity Click here for additional data file. Copyright: © 2017 Tchokponhoué DA et al. 2017 Data associated with the article are available under the terms of the Creative Commons Zero "No rights reserved" data waiver (CC0 1.0 Public domain dedication).

Fruiting intensity and correlation between growth parameters and fruiting Click here for additional data file. Copyright: © 2017 Tchokponhoué DA et al. 2017 Data associated with the article are available under the terms of the Creative Commons Zero "No rights reserved" data waiver (CC0 1.0 Public domain dedication).

**Table 6.** Correlation matrix of vegetative growth and development parameters in *Synsepalum dulcificum*'s juveniles.

	Stem collar diameter	Height	Number of branches	Number of leaves	Leaf area	Number of fruits
Stem collar diameter						
Height	0.81***					
Number of branches	0.69***	0.66***				
Number of leaves	0.84***	0.74***	0.75***			
Leaf area	0.68***	0.53***	0.47***	0.61***		
Number of fruits	0.57***	0.59***	0.54***	0.7***	0.34*	

* Significant at 5%, ** = Significant at 1%, ***= Significant at 1‰

### Phenophases length

**Figure 5.**
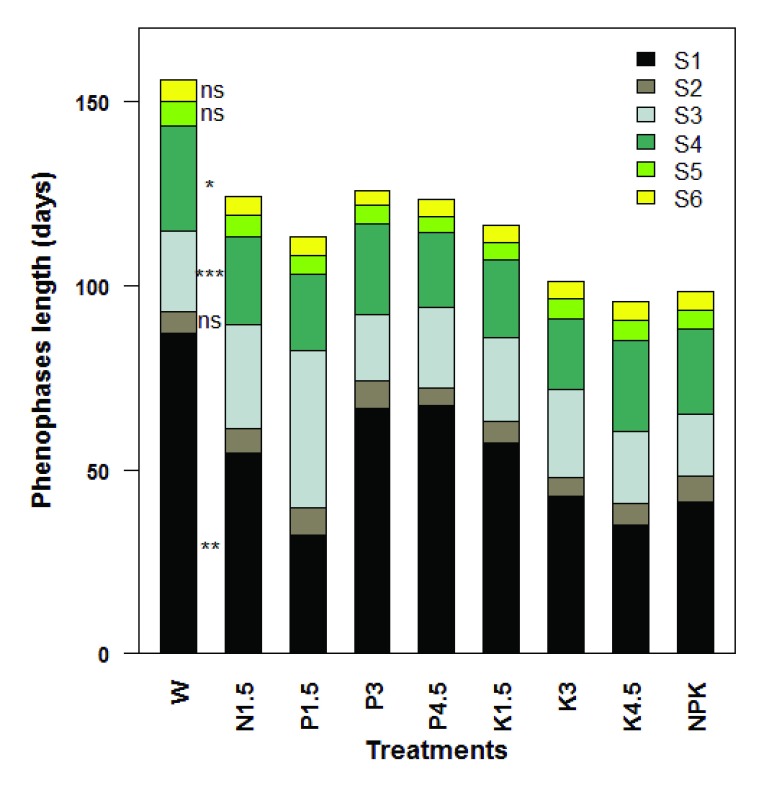
Phenophases duration in juveniles of *Synsepalum dulcificum* under various treatments (n = 5 – 10 seedlings). ( **S _1_**) Time from budding to flowering; ( **S _2_**) Time from flowering to flower bloom; ( **S _3_**) Time from flower bloom to fructification onset; ( **S _4_**) Time from fructification onset to physiological maturity; ( **S _5_**) Time from physiological maturity to fruit ripening onset; ( **S _6_**) Time from fruit ripening onset to full ripening. ^ns^ = not significant, ^*^ Significant at 5%, ^**^ = Significant at 1%, ^***^= Significant at 1‰. W = Daily watering, no exogenous nutrients; N1.5 = Daily watering + 1.5 g N [seedling] ^-1^; N3 = Daily watering +3 g N [seedling] ^-1^; N4.5 = Daily watering + 4.5 g N [seedling] ^-1^; P1.5 = Daily watering + 1.5 g P [seedling] ^-1^; P3 = Daily watering + 3 g P [seedling] ^-1^; P4.5 = Daily watering + 4.5 g P [seedling] ^-1^; K1.5 = Daily watering +1.5 g K [seedling] ^-1^; K3 = Daily watering + 3 g K [seedling] ^-1^; K4.5 = Daily watering + 4.5 g K [seedling] ^-1^; NPK = Daily watering + 1.5 g N + 1.5 g P + 1.5 g K [seedling] ^-1^.

The lengths of the various phenophases observed during the reproductive growth of *S. dulcificum* are presented in Figure 5. The effect of treatments on the times from budding to flowering (S _1_), from flower bloom to fructification onset (S _3_), and from fructification onset to physiological maturity (S _4_) were very significant ( *P <* 0.01), highly significant ( *P <* 0.001) and significant ( *P <* 0.05), respectively. The shortest length for S _1_ was observed in juveniles fertilized with 1.5 g phosphorus (32.33 ± 6.97 days), whereas the longest time for S _1_ was recorded in daily watered unfertilized juveniles (87.00 ± 12.52 days). NPK-fertilized juveniles rapidly started fruiting (within 16.66 ± 3.32 days), once their flowers bloomed. The longest time from fructification onset to physiological maturity (S _4_) was recorded in daily watered unfertilized juveniles (W) (28.66 ± 3.52 days).

Phenophase length Click here for additional data file. Copyright: © 2017 Tchokponhoué DA et al. 2017 Data associated with the article are available under the terms of the Creative Commons Zero "No rights reserved" data waiver (CC0 1.0 Public domain dedication).

### Relationship between growth traits and fruit production

The correlation matrix overall indicated positive and highly significant correlation between growth traits; a higher correlation was observed between the stem collar diameter and the number of leaves ( Table 6). Similarly, correlations between fruit production and growth traits are all positive but higher with leaves production than other growth traits. The regression equation for fruit production in juveniles reads: ***ln (Number of fruit) = -4.51 + 1.15 ln (Number of leaves)***.

## Discussion

### Growth and reproductive responses of *S. dulcificum* seedling to watering and fertilization treatments

In *S. dulcificum*’s juveniles the use of appropriate fertilizer at a relevant dose is critical to avoid detrimental effects. The present study showed that while seedlings with phosphorus and potassium supply maintained survival at a high rate, nitrogen fertilization decreased survival rate with an increasing prevalence of dead seedlings as the dose increased. Similar negative effects of a larger nitrogen supply on survival was also reported in *Trifolium medium* L. ( Chmelíková & Hejcman, 2014) and in *Eucalyptus pauciflora* Sieber ex Sprengel ( Atwell *et al.*, 2009). Likewise, in *Betula pubescens* Ehrh., *Larix sibirica* Ledeb., and *Picea sitchensis* (Bong.) Carr seedlings fertilized with nitrogen at the rate of 3.7 g [seedling] ^-1^ had lower survival than those fertilized with 1.2 g [seedling] ^-1^ ( Oskarsson *et al.*, 2006). Therefore, for 15 month-old juveniles of *S. dulcificum* we should limit the nitrogen dose to 1.5 g [seedling] ^-1^ to encourage further growth and development.

Juvenility represents a crucial stage in survival, functional and productive traits of plant species ( Trubat *et al.*, 2010), and improving the performance of plant species at this stage through fertilization is desirable. Though the beneficial effect of fertilization on juveniles of tree species is questionable ( Akinnifesi *et al.*, 2008; Ebert *et al.*, 2002), our results revealed that in the case of *S. dulcificum,* all vegetative growth traits positively responded to water supply and fertilization. We observed two main morphotypes in juveniles of *S. dulcificum* in response to treatments. The first morphotype was ‘ *thin’* and exclusively observed in the field where juveniles were rain fed, and where the plant mainly grew in height as an adaptation strategy to cope with weed competition for the light and gained a limited number of branches and leaves. In contrast, when water and/or nutrients were supplied, this induced a ‘ *well-branched’* morphotype. The characteristics of this morphotype included a high stem collar diameter, a high number of leaves and branches and a dense crown. NPK application to 15 months old seedlings improved vegetative growth. For instance, at the end of the experiment, initial stem collar diameter and leaf number increased by 1.6 fold and 18 folds, respectively, in 15-month old juveniles watered and supplied with NPK; whereas in control juveniles (without watering and fertilization), initial stem collar diameter, height, and number of leaves increased by 1.36 fold and 6.41 folds, respectively. This performance of NPK-fertilized seedlings highlighted the additive effect of those three nutrients (N, P and K) ( Chang, 2003).

At 28 months old, juveniles were 47 cm tall after 13 months of fertilization with a 23.2 cm gain. Existing literature reported that the species height at four years old was 50–60 cm ( Joyner, 2006). Even under a fertilization regime, *S. dulcificum* height growth did not dramatically improve, particularly compared to other tropical fruit species, such as *Vitex doniana* Sweet in which seedlings in nursery reached 75 cm before one year old ( N’Danikou *et al.*, 2015). However, the effect of NPK on the vegetative growth was reflected in increased branch and leaf numbers, which represents an interesting prerequisite to further investigation of the species’ response to increased dose of the N, P, and K combination.

More importantly, our findings provided evidence (for the first time) of the beneficial effect of water supply and fertilization on *S. dulcificum* flowering and fructification *.* Only juveniles that were daily watered entered in the generative phase. No bud and flower were observed in juveniles evolving in natural conditions, i.e. rain-fed juveniles. This suggested water supply as the key determinant for *S. dulcificum* juveniles’ entrance into reproductive phase. This finding is in line with Bernier *et al.* (1993) who indicated that any environmental factors that have the ability to change regularly (e.g. photoperiod, temperature, water availability) can control plant development towards flowering. While perennial species were reported to exhibit, in general, a long juvenile phase ( Hanke *et al.*, 2007) that could reach up to five years (e.g. *Olea europea* L., *Malus domestica* Borkh.) ( Santos-Antunes *et al.*, 2005; Zimmerman, 1972), this juvenile phase (ending with budding) can be shortened in *S. dulcificum* from > 36 months to 21 months with simple daily water provision. Our results also revealed that when suitable fertilization scheme was combined to daily watering, first flowering occurred in *S. dulcificum* at an average age of 23 months (less than two years old) and at 16 months old for early flowering individuals. This highlighted the importance of nutrient balance to the development of fruit tree species. First fruiting occurred at the average age of 24 months (20 months for extra early individuals). This achievement represented a major progress in the improvement of the species reproduction, as previous reports indicated that *S. dulcificum* bears fruit after 3 to 4 years ( Joyner, 2006). Although water supply was crucial for *S. dulcificum* to initiate generative phase, our findings also suggested that nutrient supply is of paramount importance for the species productivity. This is illustrated by the fruit production that is fivefold higher in juveniles receiving NPK in addition to daily watering than in juveniles that benefited just of daily watering.

Our findings also expand the current knowledge on the phenology and reproductive biology of *S. dulcificum*. In juveniles of *S. dulcificum,* budding is continuous once it started, provided water is available. Flowering occurred one to three months after budding. In the first production round, flower production started from within the crown outward. This same “centrifugal” flowering pattern was also reported in *Acer platanoides* L. ( Tal, 2011). Flower bloom occurred five to seven days after flowering and was always observed at the hot hours of the day (from 11 a.m. to 4 p.m.). In this study, we observed that flowers fully exposed to sun bloomed quicker than those hidden in the plant crown. This was well observed in NPK-fertilized seedlings and we suspected the flower bloom time in *S. dulcificum* to be light-dependent. This suspicion could even be expanded to the whole reproductive stage length of the species, since Xingway & Abdullah (2016) reported that four year old juveniles kept under shelter took 200 days from budding to fruiting stage, whereas in this study, sun exposed juveniles fruited within 100 – 160 days after budding. The growth stage also played a key role in the length of *S. dulcificum* phenophases. In adult trees, the timeframe from flowering to fruiting was estimated at seven days ( Oumorou *et al.*, 2010), while in juveniles, we observed that flowering to fruiting lasted 46 to 57 days.

### Implications for crop improvement and increased production

*S. dulcificum* as a sweetener and source of secondary metabolites has a lot of potential as a future crop that can be used to reduce the prevalence of diabetes, high blood pressure, and other diseases due to inadequate nutrition. The species has suffered from lack of interest and is rarely included in breeding programs. Moreover, strategies to develop cultivars are still obscure. Also, agronomic practices to improve production and seed management require increased mobilization of resources. Our study is the first of its kind, and reports on the effect of water and nutrient management on flowering and fruiting in *S. dulcificum*. When the suitable nutrient was combined to regular water supply, fructification time in *S. dulcificum* can be reduced to half of its natural duration.

Inorganic fertilization significantly improved *S. dulcificum* growth; however, the most efficient fertilizer formulation is yet to be determined. Moreover, the use and the effects of organic fertilization on the species growth and fruit production should be explored. A major reason of the renewed interest in *S. dulcificum* is its high content in secondary metabolites. In our study, the effect of fertilization on metabolite content was not assessed and future studies should shed light on that effect, as well as on the metabolite production across ecological gradients.

To date only limited knowledge is available on the genetic variation in *S. dulcificum* and the distribution of genotypes across Africa. *S. dulcificum* is reported to be native to West Africa and thrives in Ghana, Benin, Togo, and Nigeria. Assessment of the genetic diversity and the definition of heterotic groups, as well as a region-wide collection of germplasms, are necessary to gather ecotypes and cultivars to increase the range of diversity and enable the development of breeding populations.

*S. dulcificum* is a shrub that naturally matures after three to four years. Although regular watering and nutrient supply can accelerate fruit production, it will be useful to identify secondary traits related to yield so as to increase predictive accuracy and efficient breeding plan (e.g. efficient time management, selection of high-yielding population). In this regard, leaf production represents an interesting secondary trait to consider in correlative selection of high yielding genotypes. In our study, high leaf production was positively correlated with higher fruit production. To increase the accuracy of the selection programme, the use of quantitative traits loci might be an option. So far there are no data on genes involved in leaf and fruit production. The sequencing of the species’ genome could then enable rapid identification of such genes and other useful ones so as to strengthen the development of cultivar and the economic return of the species.

Heat and drought stresses are yet to be assessed in *S. dulcificum*. Empirical observation from the first and last authors revealed that shaded seedlings were more vigorous than sun-exposed ones. Understanding how various genotypes of *S. dulcificum* respond to environmental stresses will shed light onto which cultivar would be appropriate to which locations and help adapt to climate changes. In addition, juveniles submitted to rainfall survived as well as those regularly watered. Such a response opens room for the investigation of the adaptation potential of the species to drier environments and the side-effects of such adaptation on cultivar selection.

Phenology data presented in this study remains incomplete since it did not cover the whole year. A follow up experiment will be necessary to provide a wider view on the phenological timeframe, including analysis of the fructification frequency, the period of flowering and fructification peak, and their variation across dry and rainy reasons.

## Conclusions

This study has highlighted the beneficial effect of water supply and fertilization on both vegetative and reproductive growth in *S. dulcificum*. Water supply appeared as the most important factor unlocking flowering in the species, while nutrient supply was crucial in accelerating entrance into reproductive phase and enhancing fruit production. Throughout the experiment, the combination of nitrogen, phosphorus and potassium at the dose of 1.5 g (for all) consistently exhibited the highest performance for all growth and yield traits. These findings represent a crucial progress towards the species breeding and production scaling up.

## Data availability

The data referenced by this article are under copyright with the following copyright statement: Copyright: © 2017 Tchokponhoué DA et al.

Data associated with the article are available under the terms of the Creative Commons Zero "No rights reserved" data waiver (CC0 1.0 Public domain dedication). 

**Dataset 1. Initial growth parameters at the fertilization experiment onset.** D0 = Initial diameter, H0 = Initial height, L0 = Initial number of leaves, and B0 = Initial branching. This dataset was used to prepare Table 2. doi, 10.5256/f1000research.11091.d155614 ( Tchokponhoué *et al.*, 2017a)

**Dataset 2. Survival data.** This dataset was used to prepare Figure 2 and Table 3 and to perform related analysis. “Status” refers to whether the seed died (1) or was still alive at the end of the experiment (0) and “Time” refers to the number of weeks after each the seedling died (for dead seedlings) or the last time we saw surviving seedling (for seedlings still alive at the end of the experiment). doi, 10.5256/f1000research.11091.d155615 ( Tchokponhoué *et al.*, 2017b)

**Dataset 3. Growth parameters (increment) at the end of the experiment for vegetative growth.** This dataset was used to prepare Figures 3A–D and to perform related analysis. doi, 10.5256/f1000research.11091.d155616 ( Tchokponhoué *et al.*, 2017c)

**Dataset 4. Growth parameters at the end of the experiment for leaf area.** This dataset was used to prepare Figure 3E and to perform related analysis. doi, 10.5256/f1000research.11091.d155626 ( Tchokponhoué *et al.*, 2017d)

**Dataset 5. Reproductive performance (time to budding).** This dataset was used to prepare Figure 4A and to perform related analysis. doi, 10.5256/f1000research.11091.d155627 ( Tchokponhoué *et al.*, 2017e)

**Dataset 6. Reproductive performance (time to flowering).** This dataset was used to prepare Figure 4B and to perform related analysis. doi, 10.5256/f1000research.11091.d155628 ( Tchokponhoué *et al.*, 2017f)

**Dataset 7. Reproductive performance (time to fruiting).** This dataset was used to prepare Figure 4C and to perform related analysis. doi, 10.5256/f1000research.11091.d155629 ( Tchokponhoué *et al.*, 2017g)

**Dataset 8. Cumulative fruiting.** This dataset was used to prepare Figure 4D and to perform related analysis. doi, 10.5256/f1000research.11091.d155630 ( Tchokponhoué *et al.*, 2017h)

**Dataset 9. Budding intensity.** This dataset was used to prepare Table 5 and to perform related analysis. doi, 10.5256/f1000research.11091.d155631 ( Tchokponhoué *et al.*, 2017i)

**Dataset 10. Fruiting intensity and correlation between growth parameters and fruiting.** This dataset was used to prepare Table 5 and to generate Table 6 (correlation matrix), and to perform related analysis. doi, 10.5256/f1000research.11091.d155632 ( Tchokponhoué *et al.*, 2017j)

**Dataset 11. Phenophase length.** This dataset was used to generate Figure 5 and to perform related analysis. doi, 10.5256/f1000research.11091.d155633 ( Tchokponhoué *et al.*, 2017k)
