# Supplementary material for: Early fruiting in Synsepalum dulcificum (Schumach. & Thonn.) Daniell juveniles induced by water and inorganic nutrient management
Source: F1000Res. 2017 Mar 30;6:399. [Version 1] doi: 10.12688/f1000research.11091.1 (PMC5461899; doi:10.12688/f1000research.11091.1)
Supplement: Peer review history

# Peer review: Referee response for version 1

**DOI:** 10.5256/f1000research.11963.r23248

**Published:** 2017-06-05

**Authors:** Nur Ashikin Psyquay Abdullah

**Affiliations:** 1 Department of Crop Science, Faculty of Agriculture and Food Science, Universiti Putra Malaysia, Sarawak, Malaysia

When the title presented was as “induced by water and inorganic nutrient”, I was expecting different watering different regimes. It comes to no surprise that the control will have least significant effects on the plant growth since it received no fertilizers. If the authors would have applied the same fertilizing regimes on the rainfall fed, then they would have stronger arguments that water did influence the onset of flowering. The same opinion is applied to the treatments in the glasshouse. Obviously, no watering on fertilized plants will gives devastating effects on the seedlings. However, if they would had expand the watering regimes to different volumes such as 1,2 and 3 L for example, giving some stress induced conditions to the seedling then perhaps they could gives a strong conclusion that watering was the main effects in inducing flowering. Their arguments fall towards more on the watering rather than the fertilizing as this comprise the main treatments. The results presented with comment “Finding providing evidence of the beneficial effect of water supply and fertilizing (for the first time)” for me is hardly surprising or new. Many plants that are put into cultivation must go through cultivation studies to determine the optimum agronomic practices, and basically water and nutrients are beneficial to obtained optimum yield. What I would like the author to stress on, since few studies are put into the cultivation of miracle fruit is the emphasis on the best fertilizing regimes for its growth. For me, by chance the 2L water did induced flowering as compared to the rain fed plants. In my opinion, the rain should had been measured. In the future, when this plant is plant in the field, how will they expect to provide 2L water or are this recommended for indoor pot plant?

 Nevertheless, this paper is recommendable since we need as much baseline data for the cultivation of this underutilized plant.

I have read this submission. I believe that I have an appropriate level of expertise to confirm that it is of an acceptable scientific standard.

# Peer review: Referee response for version 1

**DOI:** 10.5256/f1000research.11963.r21888

**Published:** 2017-04-26

**Authors:** Emil Luca, Laura Cristina Luca

**Affiliations:** 1 Department of Horticulture and Landscaping, University of Agriculture and Veterinary Medicine, Cluj-Napoca, Romania; 2 University of Agriculture and Veterinary Medicine, Cluj-Napoca, Romania

It was a great and pleasant surprise for me to read such a well-documented paper. The results obtained by the authors are revealing the intensive research conducted in the almost 3 years of experiments, and those results are properly highlighted in the content of the present paper.

 The documentation was also very detailed and thoroughly done, which demonstrates the authors' involvement in the chosen topic, as well as their dedication to it.

 Through their research, the authors managed to shorten the juvenile phase (ending with budding) at *Synsepalum dulcificum* from more than 36 months to 21 months with simple daily water provision, while when suitable fertilization scheme was combined to daily watering, first flowering occurred at an average age of 23 months and at 16 months old for early flowering individuals. In the article it is also stated that the first fruiting occurred at the average age of 24 months. These achievements shorten almost to half the vegetation period for *Synsepalum dulcificum, *which means that this miraculous plant has the chances to be bred and produced on a large scale very soon.

 My hope is that the authors will continue their research, for in the near future we would be able to find a way to produce it in other parts of the world.

We have read this submission. We believe that we have an appropriate level of expertise to confirm that it is of an acceptable scientific standard.
